# Machine learning prediction of obesity-associated gut microbiota: identifying *Bifidobacterium pseudocatenulatum* as a potential therapeutic target

**DOI:** 10.3389/fmicb.2024.1488656

**Published:** 2025-02-05

**Authors:** Hao Wu, Yuan Li, Yuxuan Jiang, Xinran Li, Shenglan Wang, Changle Zhao, Ximiao Yang, Baocheng Chang, Juhong Yang, Jianjun Qiao

**Affiliations:** ^1^Zhejiang Institute of Tianjin University (Shaoxing), Shaoxing, China; ^2^NHC Key Lab of Hormones and Development and Tianjin Key Lab of Metabolic Diseases, Tianjin Medical University Chu Hsien-I Memorial Hospital & Institute of Endocrinology, Tianjin, China; ^3^Yidu Cloud (Beijing) Technology Co., Ltd., Beijing, China; ^4^Department of Pharmaceutical Engineering, School of Chemical Engineering and Technology, Tianjin University, Tianjin, China; ^5^Guangdong Medical University, Zhanjiang, China

**Keywords:** overweight, machine learning, XGBoost-SHAP, intestinal microbiota, *Bifidobacterium pseudocatenularis*

## Abstract

**Background:**

The rising prevalence of obesity and related metabolic disorders highlights the urgent need for innovative research approaches. Utilizing machine learning (ML) algorithms to predict obesity-associated gut microbiota and validating their efficacy with specific bacterial strains could significantly enhance obesity management strategies.

**Methods:**

We leveraged gut microbiome data from 1,563 healthy individuals and 2,043 overweight patients sourced from the GMrepo database. We assessed the anti-obesity effects of *Bifidobacterium pseudocatenulatum* through experimentation with *Caenorhabditis elegans* and C3H10T1/2 cells.

**Results:**

Our analysis revealed a significant correlation between gut bacterial composition and body weight. The top 40 bacterial species were utilized to develop ML models, with XGBoost demonstrating the highest predictive accuracy. SHAP analysis indicated a negative association between the relative abundance of six bacterial species, including *B. pseudocatenulatum*, and body mass index (BMI). Furthermore, *B. pseudocatenulatum* was shown to reduce lipid accumulation in *C. elegans* and inhibit lipid differentiation in C3H10T1/2 cells.

**Conclusion:**

*Bifidobacterium pseudocatenulatum* holds potential as a therapeutic agent for managing diet-induced obesity, underscoring its relevance in microbiome-based obesity research and intervention.

## Introduction

Overweight and obesity are major global public health challenges, presenting substantial clinical difficulties (Laine and Wee, 2023). Obesity is a key precursor to various widespread diseases, including type 2 diabetes, hypertension, non-alcoholic fatty liver disease, cancer, and obstructive sleep apnea syndrome (Tsai and Bessesen, 2019; Quek et al., 2023). Despite numerous efforts aimed at treatment, mitigation, and prevention, the global prevalence and severity of obesity continue to rise (Heindel et al., 2024). Etiologically, obesity is a complex condition driven primarily by the interplay of genetic and environmental factors (Laine and Wee, 2023). As a result, effective obesity treatment requires a multidisciplinary approach (Velazquez and Apovian, 2018), highlighting the urgent need for innovative prevention and therapeutic strategies.

The gut microbiota plays a crucial role in the digestion, absorption, and metabolism of food, influencing body weight through the regulation of metabolism, appetite, bile acid metabolism, and the endocrine, nervous, and immune systems (Van Hul and Cani, 2023). Studies by Cho et al. (2012) and Cox et al. (2014) demonstrated that subtherapeutic doses of antibiotics in mice lead to increased weight and fat mass, underscoring the gut microbiota’s role in the etiology of obesity. Backhed et al. (2004) further showed that gut microbiota directly participate in host energy metabolism; colonizing germ-free mice with gut microbiota increases blood glucose and insulin levels while promoting lipid storage in adipose tissue. Research also indicates that omega-3 fatty acids protect against mild inflammation, metabolic endotoxemia, insulin resistance, and obesity. Probiotics such as *Bifidobacterium*, *Lactobacillus*, and *Akkermansia muciniphila* have been associated with the beneficial effects of omega-3 (Carvalho et al., 2012). Although there is no conclusive evidence that specific microbes are the primary cause of obesity or overweight issues, the role of gut microbiota in metabolic disorders and obesity has been consistently demonstrated (Liang et al., 2023). Notably, various probiotic strains have been shown to effectively reduce BMI (Tome-Castro et al., 2021), with significant research focusing on *Lactobacillus*, *Bifidobacterium*, and *Akkermansia muciniphila*, which can improve obesity, blood glucose levels, and insulin resistance (Koutnikova et al., 2019; Qiu et al., 2022; Dao et al., 2016). Thus, the targeted study of gut microbiota as early indicators and preventive measures for obesity, along with the development of probiotics with potential anti-obesity effects, is vital for effective obesity management.

Given the numerous interfering factors and significant challenges in detecting gut microbiota, the lack of objective and large-scale clinical data is likely a key reason why gut microbiota have struggled to predict overweight and obesity. In this context, machine learning (ML) offers a promising approach by classifying the gut microbiota of normal and overweight populations based on extensive clinical datasets. ML algorithms, rather than solving inference problems or understanding the relationships between variables, learn directly from data, identifying complex nonlinear patterns to make accurate predictions (Deo, 2015). By exploring disease risk through ML, we can obtain quick, real-time, and interpretable results regarding risk factors and conditions (Safaei et al., 2021). Therefore, this study aims to use feature selection methods to characterize the gut microbiota landscape, develop and validate ML algorithms to identify overweight individuals, and apply interpretability techniques to visualize predictive factors. Additionally, by leveraging ML methods to discover potential probiotics, we validate the anti-obesity effects of specific strains through nematode and cell experiments, offering a new perspective for probiotic development.

## Materials and methods

### Data sources and processing

The human gut microbiota data for this study were obtained from the GMrepo database,^1^ including individuals aged 18 years and older classified as either healthy or overweight. Overweight was defined as BMI ≥25, encompassing individuals with BMI ≥30 (commonly classified as obese). To ensure homogeneity, samples from individuals with other underlying diseases were excluded, and participants were categorized into two groups: overweight (BMI ≥25) and control (BMI <25). Stratified sampling was applied to the overweight group to minimize biases related to age and gender distribution, resulting in 3,606 samples analyzed (2,043 overweight and 1,563 control). The inclusion of individuals with obesity under the overweight category is discussed as a potential limitation. Data were imported into Python using pandas, enabling operations like filtering, alignment, and transformation. Missing values for bacterial relative abundance and gender were replaced with zeros to ensure data integrity. *Z*-score normalization was applied within cross-validation to avoid data leakage by calculating normalization parameters only on the training data and applying them to the test data. Samples with low sequencing coverage (<1%) were excluded, and potential contaminants were identified and removed through background sample comparisons, ensuring a robust and high-quality dataset.

### Microbiota diversity indices

We calculated the Shannon index and Chao1 index to assess the diversity of gut microbiota among the study groups. The Shannon index provides insights into the richness and evenness of microbial communities, while the Chao1 index estimates species richness based on the number of observed species and their frequency.

### Statistical analysis and visualization

To visualize the microbial diversity and community structure, we generated heatmaps and boxplots. Heatmaps illustrate the relative abundance of microbial taxa across samples, highlighting patterns of similarity and differences between groups. Boxplots provide a comparative overview of diversity indices between the overweight and control groups, facilitating the identification of significant differences.

### Data standardization

In this study, we applied *z*-score standardization to all input features to ensure that the features were on the same scale for comparison. The standardization was performed before model training to eliminate differences in scale between features, preventing certain features from disproportionately influencing the model results due to their different units. All numerical data (such as microbial community relative abundance and sample information) were standardized before being input into the machine learning models. This step helped improve the model’s stability and ensured that all features had equal weight during model training.

### Model construction and evaluation

To investigate the potential of gut microbiota dysbiosis as biomarkers for overweight, we developed machine learning models using XGBoost, support vector machine (SVM), logistic regression, and decision tree algorithms. The optimization target for model performance was AUC (area under the ROC curve), though additional metrics such as accuracy, precision, recall, and *F*_1_-score were also assessed. For each algorithm, we used the following settings: In XGBoost, hyperparameter optimization was performed using GridSearchCV, exploring the ranges of colsample_bytree: [0.8, 0.9, 1.0], gamma: [0, 0.1, 0.2], learning_rate (eta): [0.01, 0.1, 0.2], max_depth: [3, 4, 5], n_estimators: [50, 100, 150], and subsample: [0.8, 0.9, 1.0]. A 5-fold cross-validation strategy was employed to prevent overfitting and ensure robust model performance. For SVM (support vector machine), the radial basis function (RBF) kernel was used, and hyperparameters such as *C* (regularization parameter) and gamma (kernel coefficient) were optimized using a grid search. The values tested for *C* ranged from 1 to 100, and gamma ranged from 0.001 to 0.1. In Logistic Regression, default parameters were used, with the main hyperparameter being the regularization parameter *C*, which was optimized using GridSearchCV with values from 0.01 to 10. For the Decision Tree model, the default max_depth parameter was used, and different values for min_samples_split (ranging from 2 to 10) and min_samples_leaf (ranging from 1 to 4) were explored to control tree growth and avoid overfitting. After comparing performance metrics, including AUC, we determined that XGBoost was the most effective model for predicting overweight status based on gut microbiota data.

### SHAP-based model interpretability analysis

To interpret the XGBoost model’s predictions, we employed SHAP (Shapley Additive Explanations), an approach grounded in game theory (Li et al., 2023). SHAP quantifies the contribution of each feature to the model’s output by assigning a Shapley value that indicates the direction and magnitude of each feature’s impact. Positive SHAP values (>0) suggest a positive influence on the prediction, while negative SHAP values (<0) indicate a negative influence (Song et al., 2023). SHAP provides both global and local insights: Global Interpretation calculates the average SHAP value of each feature across all samples, highlighting overall feature importance, while local interpretation assesses each feature’s impact on individual predictions. The SHAP framework enabled us to generate “SHAP Summary Plots,” visualizing feature importance and contributing to a deeper understanding of the influence of gut microbiota features on overweight classification.

### Bacterial culture and co-culture experiments

We cultured *B. pseudocatenulatum* strains (JXL-01, JXL-02, JXL-03, and JXL-05) anaerobically in MRS medium (Shanghai Macklin Biochemical Technology Co., Ltd.) at 37°C for 24 h. Once the bacterial concentration reached 10^9 CFU/mL, the culture was centrifuged at 7,000 rpm for 15 min. The supernatant was discarded, and the bacterial pellet was washed several times with sterile PBS. The bacteria were then resuspended in complete DMEM medium and incubated anaerobically at 37°C for an additional 2 h. The supernatant was collected for subsequent cell experiments.

### Synchronization of *Caenorhabditis elegans*

L4-stage *C. elegans* were collected in a centrifuge tube, and 1 mL of M9 buffer was added to wash away excess *E. coli* OP50. After allowing the worms to settle, the supernatant was removed, and this washing step was repeated three times. Next, 1 mL of lysis solution was added and thoroughly mixed. After discarding the supernatant, the worms were washed three times with M9 buffer via centrifugation (3,000 rpm, 1 min), retaining the eggs. The eggs were then transferred to new NGM plates and incubated at 20°C in a biochemical incubator. After approximately 16–18 h, the eggs developed into L1-stage larvae.

### Culture of *Caenorhabditis elegans* and fat measurement

*B. pseudocatenulatum* strains (JXL-01, JXL-02, JXL-03, and JXL-05) and *E. coli* OP50 were cultured in liquid medium until reaching the logarithmic phase. After centrifugation at 8,000 rpm for 10 min at 4°C, the bacterial pellets of *B. pseudocatenulatum* were collected and resuspended in M9 buffer to an OD of 0.5. A total of 100 μL of the bacterial suspension was then applied to 60 mm NGM solid plates containing the worm eggs and incubated at 20°C. After approximately 3 to 4 days, the worms developed into L4-stage larvae, with *E. coli* OP50 serving as a control group. Following an additional 3 days of continued culture with the bacterial suspension, 20 randomly selected worms were subjected to Oil Red O staining to measure fat accumulation. The worms were rinsed three times with M9 buffer, anesthetized with 25 mM levamisole hydrochloride, and treated with 200 μL of 4% paraformaldehyde solution for 20 min. The solution was removed, and the worms underwent three freeze–thaw cycles using liquid nitrogen. Subsequently, the worms were stained with 60% isopropanol and Oil Red O solution for 5 h. Excess dye was rinsed off with M9 buffer, and the staining was observed under an optical microscope to analyze fat content.

### Culture and differentiation of C3H10 into adipocytes

Mouse mesenchymal stem cells (C3H10 cells) were generously provided by Professor En-Dong Zhu’s research group at the Endocrinology Institute of Tianjin Medical University. The cells were cultured in DMEM medium (Gibco, United States) at 37°C, supplemented with 10% fetal bovine serum (FBS) (Hyclone, United States) and 100 U/mL penicillin/streptomycin (Beijing ZSbio). They were divided into two groups: a control group and a *B. pseudocatenulatum* metabolite intervention group (B.p), with the intervention lasting for 24 h. Following this, the cells were treated with specific differentiation media to induce differentiation into adipocytes. Differentiation Induction Agent A, consisting of DMEM high-glucose complete medium supplemented with 0.2 μg/mL dexamethasone, 5 μg/mL insulin, 100 μM indomethacin, and 250 μM IBMX, was applied for 3 days. This was followed by Differentiation Induction Agent B, which included DMEM high-glucose complete medium with 5 μg/mL insulin, administered for an additional 3 days. This differentiation process ensured that C3H10 cells functionally mimic adipose tissue, making them suitable for subsequent fat accumulation studies. For co-culture experiments, we used 10% of the metabolites from the bacterial cultures, ensuring that the conditions for cell differentiation were consistent across all experiments.

### Cell viability

Cell viability was assessed using the CCK-8 Cell Counting Kit (Shanghai Taosu Bio Technology Co., Ltd.) according to the manufacturer’s instructions. C3H10 cells were seeded in a 96-well plate with 100 μL of cell suspension per well and incubated at 37°C with various concentrations of *B. pseudocatenulatum* for 24 h. Following treatment, 10 μL of CCK-8 reagent was added to each well and incubated for an additional 30 min. Absorbance at 450 nm was then measured using a multifunctional microplate reader (Thermo Fisher Scientific), and cell viability percentages were calculated for each concentration.

### Oil Red O staining and lipid droplet analysis

Oil Red O staining was performed, and lipid droplets were analyzed using cell imaging. The procedure followed the Oil Red O kit instructions (Beijing Solarbio Science & Technology Co., Ltd.). First, Solution A and Solution B were mixed in a 3:2 ratio to prepare the staining solution. Cells were fixed with ORO fixative for 25 min, followed by staining with freshly prepared Oil Red O solution for the recommended time. After staining, cells were washed to remove excess dye, and the cell nuclei were counterstained with Mayer’s Hematoxylin, followed by washing with distilled water. Finally, the cells were treated with ORO buffer for 1 min and observed under a microscope to analyze the lipid droplets.

### Quantitative reverse-transcription PCR

Total RNA was purified from cells using the Total RNA Extraction Kit (Tiangen Biotech Co., Ltd.) according to the manufacturer’s instructions. After quantifying the RNA concentration and purity, 1 μg of RNA was reverse transcribed using the M-MuLV First Strand cDNA Synthesis Kit (Tiangen Biotech). The resulting cDNA was then amplified on a PCR cycler (Eppendorf, Germany) using the SYBR Green Real-Time PCR Kit (Tiangen Biotech). Relative mRNA levels were determined using the $2^{-\Delta \Delta C_{T}}$ method, with GAPDH mRNA serving as the reference.

The primer sequences used in the study are shown in the Supplementary Table S1.

### Statistical analysis

Statistical analysis in this study was conducted using several methods. ML analysis was performed using Python (version 3.10.2), while the Mann–Whitney U test was applied for non-parametric data, with *p*-values adjusted using the Bonferroni method and a significance level set at *p* < 0.01. Experimental data were analyzed using SPSS (version 25.0). Data normality was assessed, and for normally distributed data, independent samples *t*-tests were used to compare two groups. Results were expressed as mean ± standard deviation (SD), with a *p*-value of *p* < 0.05 considered statistically significant. Data processing for image analysis was carried out using Fiji ImageJ. These methods ensured rigorous and accurate data analysis, providing reliable and meaningful results for the study.

## Results

### Data processing results

**Figure 1:**
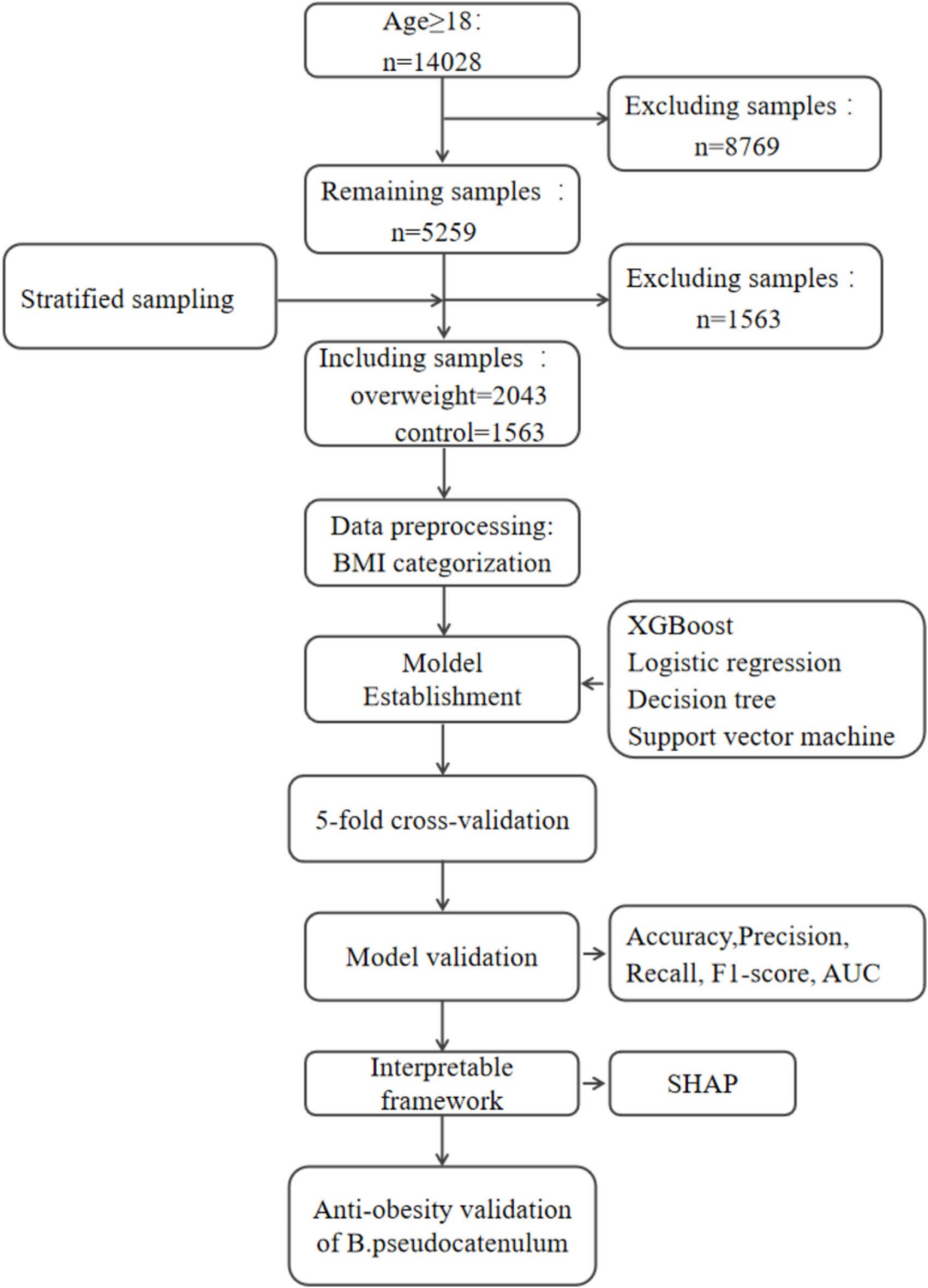
Workflow of this study. Hyperparameter tuning was conducted exclusively on training data. Validation data was only used for performance evaluation to avoid data leakage. XGBoost, extreme gradient boosting; LR, logistic regression; DT, decision tree; SVM, support vector machine; SHAP, SHapley Additive exPlanations.

From the GMrepo database, a total of 14,028 samples containing human gut microbiome information were downloaded. After sample screening and processing, samples that did not meet the criteria were excluded, leaving 5,259 samples. Using BMI ≥25 as the threshold for overweight classification, the samples were divided into an overweight group and a control group. Further stratified screening of the samples resulted in the final inclusion of 3,606 samples, with 1,563 in the normal group and 2,043 in the overweight group, as illustrated in Figure 1 and detailed in Supplementary Table S1.

The study included a total of 4,419 bacterial species. Analysis of the gut microbiota in the two sample groups revealed that the relative abundance and frequency of 705 bacterial species differed significantly between the groups (*p* < 0.01). After applying Bonferroni correction, 283 bacterial species remained significantly different (adjusted *p*-value <0.01). These bacterial species were then ranked by frequency of occurrence. Considering model complexity and computational power, the top 40 bacterial species were selected as features for constructing the ML model (Table 1). Additionally, to enhance prediction accuracy, gender and age were included as feature variables, given their potential influence on the gut microbiome composition (Hales et al., 2018; Ge et al., 2024).

**Table 1:** Top 40 target bacteria.

Species	Fisher *p*-value	Adjusted Fisher *p*-value	*p*-value	Adjusted *p*-value
Blautia obeum	7.07 × 10^−26^	3.13 × 10^−22^	8.45 × 10^−27^	3.73 × 10^−23^
*Alistipes putredinis*	2.68 × 10^−22^	1.19 × 10^−18^	1.40 × 10^−18^	6.17 × 10^−15^
*Alistipes shahii*	3.97 × 10^−22^	1.75 × 10^−18^	1.98 × 10^−11^	8.76 × 10^−8^
*Dorea formicigenerans*	1.08 × 10^−25^	4.76 × 10^−22^	2.88 × 10^−15^	1.27 × 10^−11^
*Parabacteroides merdae*	8.87 × 10^−35^	3.92 × 10^−31^	3.52 × 10^−15^	1.56 × 10^−11^
*Roseburia hominis*	8.94 × 10^−13^	3.95 × 10^−9^	2.30 × 10^−10^	1.02 × 10^−6^
*Alistipes onderdonkii*	7.97 × 10^−12^	3.52 × 10^−8^	4.09 × 10^−15^	1.81 × 10^−11^
*Akkermansia muciniphila*	1.12 × 10^−38^	4.96 × 10^−35^	3.06 × 10^−46^	1.35 × 10^−42^
*Eubacterium ventriosum*	1.62 × 10^−15^	7.16 × 10^−12^	1.34 × 10^−8^	5.92 × 10^−5^
*Alistipes finegoldii*	2.05 × 10^−6^	0.009046201	8.88 × 10^−8^	0.000392558
*Clostridium leptum*	2.10 × 10^−26^	9.27 × 10^−23^	2.20 × 10^−32^	9.71 × 10^−29^
*Eubacterium ramulus*	2.87 × 10^−9^	1.27 × 10^−5^	9.87 × 10^−12^	4.36 × 10^−8^
*Bacteroides massiliensis*	1.35 × 10^−21^	5.96 × 10^−18^	5.09 × 10^−13^	2.25 × 10^−9^
*Odoribacter splanchnicus*	5.95 × 10^−14^	2.63 × 10^−10^	2.04 × 10^−8^	9.00 × 10^−5^
*Bilophila wadsworthia*	1.49 × 10^−35^	6.59 × 10^−32^	2.85 × 10^−27^	1.26 × 10^−23^
*Clostridium bolteae*	2.19 × 10^−16^	9.68 × 10^−13^	5.33 × 10^−17^	2.35 × 10^−13^
*Streptococcus thermophilus*	2.06 × 10^−10^	9.10 × 10^−7^	1.45 × 10^−8^	6.42 × 10^−5^
*Bacteroides intestinalis*	1.38 × 10^−25^	6.10 × 10^−22^	1.76 × 10^−19^	7.79 × 10^−16^
*Ruminococcus callidus*	1.42 × 10^−26^	6.26 × 10^−23^	8.77 × 10^−19^	3.87 × 10^−15^
*Clostridium symbiosum*	1.51 × 10^−24^	6.67 × 10^−21^	4.33 × 10^−21^	1.91 × 10^−17^
*Anaerotruncus colihominis*	1.32 × 10^−29^	5.81 × 10^−26^	2.48 × 10^−42^	1.09 × 10^−38^
*Holdemania filiformis*	1.14 × 10^−22^	5.03 × 10^−19^	2.30 × 10^−23^	1.01 × 10^−19^
*Bacteroides eggerthii*	3.67 × 10^−14^	1.62 × 10^−10^	6.49 × 10^−15^	2.87 × 10^−11^
*Alistipes indistinctus*	4.51 × 10^−18^	1.99 × 10^−14^	4.24 × 10^−15^	1.88 × 10^−11^
*Clostridium indolis*	1.44 × 10^−22^	6.36 × 10^−19^	6.01 × 10^−23^	2.65 × 10^−19^
*Oscillibacter valericigenes*	1.07 × 10^−20^	4.71 × 10^−17^	1.07 × 10^−27^	4.74 × 10^−24^
*Bacteroides nordii*	4.91 × 10^−7^	0.002171386	1.51 × 10^−6^	0.006665991
*Bacteroides coprocola*	2.66 × 10^−17^	1.18 × 10^−13^	3.41 × 10^−9^	1.51 × 10^−5^
Ruminiclostridium thermocellum	1.33 × 10^−20^	5.87 × 10^−17^	9.64 × 10^−29^	4.26 × 10^−25^
*Streptococcus mitis*	1.01 × 10^−16^	4.45 × 10^−13^	5.40 × 10^−18^	2.39 × 10^−14^
*Sporobacter termitidis*	6.89 × 10^−23^	3.04 × 10^−19^	1.66 × 10^−31^	7.35 × 10^−28^
*Coprococcus eutactus*	4.36 × 10^−16^	1.93 × 10^−12^	3.67 × 10^−16^	1.62 × 10^−12^
*Clostridium saccharolyticum*	6.32 × 10^−20^	2.79 × 10^−16^	8.26 × 10^−21^	3.65 × 10^−17^
*Eubacterium desmolans*	2.27 × 10^−19^	1.00 × 10^−15^	1.30 × 10^−19^	5.77 × 10^−16^
*Clostridium spiroforme*	3.24 × 10^−18^	1.43 × 10^−14^	1.71 × 10^−21^	7.56 × 10^−18^
*Blautia glucerasea*	3.58 × 10^−19^	1.58 × 10^−15^	9.11 × 10^−20^	4.03 × 10^−16^
*Barnesiella viscericola*	7.60 × 10^−32^	3.36 × 10^−28^	7.73 × 10^−41^	3.41 × 10^−37^
*Bifidobacterium pseudocatenulatum*	1.82 × 10^−14^	8.03 × 10^−11^	1.51 × 10^−12^	6.67 × 10^−9^
*Ruminococcus albus*	2.13 × 10^−20^	9.42 × 10^−17^	2.33 × 10^−24^	1.03 × 10^−20^
*Clostridium methylpentosum*	2.87 × 10^−21^	1.27 × 10^−17^	1.60 × 10^−20^	7.09 × 10^−17^

### Differences in gut microbiota diversity between overweight and control groups

**Figure 2:**
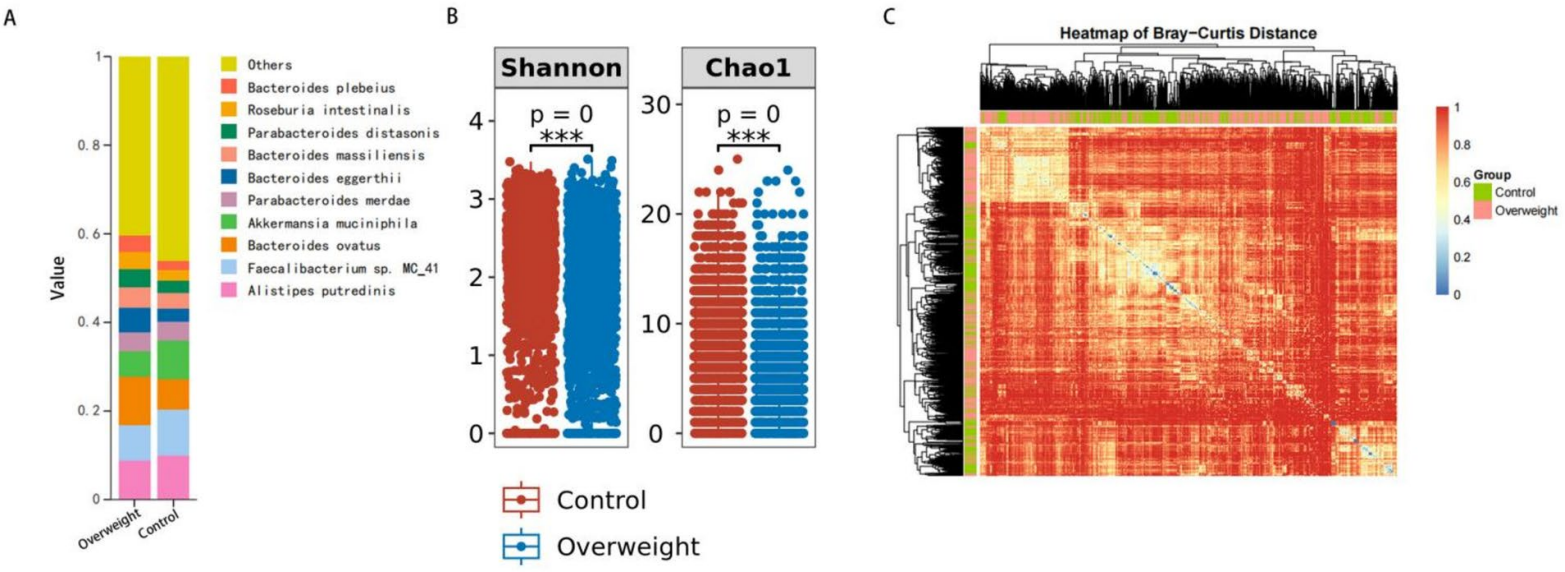
Gut microbiota diversity and composition in overweight vs. control groups. **(A)** Changes in microbial composition between overweight and control groups. **(B)** Comparison of Shannon and Chao1 diversity indices. **(C)** Clustering of samples based on microbial taxa abundance.

The analysis revealed significant differences in gut microbiota diversity between the overweight and control groups. Figure 2A presents a stacked bar chart illustrating the changes in microbial composition, emphasizing the substantial impact of overweight status on gut microbiota diversity and composition. Figure 2B displays boxplots comparing the Shannon index and Chao1 index, showing that the control group exhibited a wider range and higher median values for both indices. This further reinforces the observation of reduced microbial diversity in individuals with overweight status. Specifically, the Shannon index, which reflects the richness and evenness of microbial communities, was significantly lower in the overweight group compared to the control group. Similarly, the Chao1 index, an indicator of species richness, also demonstrated a significant decrease in the overweight group, indicating a shift in microbial diversity associated with overweight status. Figure 2C presents a heatmap generated from the relative abundance of microbial taxa, clearly illustrating the clustering of samples, with distinct groupings observed between the overweight and control groups. Specific taxa showed significant differences in abundance, supporting the findings from the diversity indices.

### XGBoost outperforms in overweight model classification

**Figure 3:**
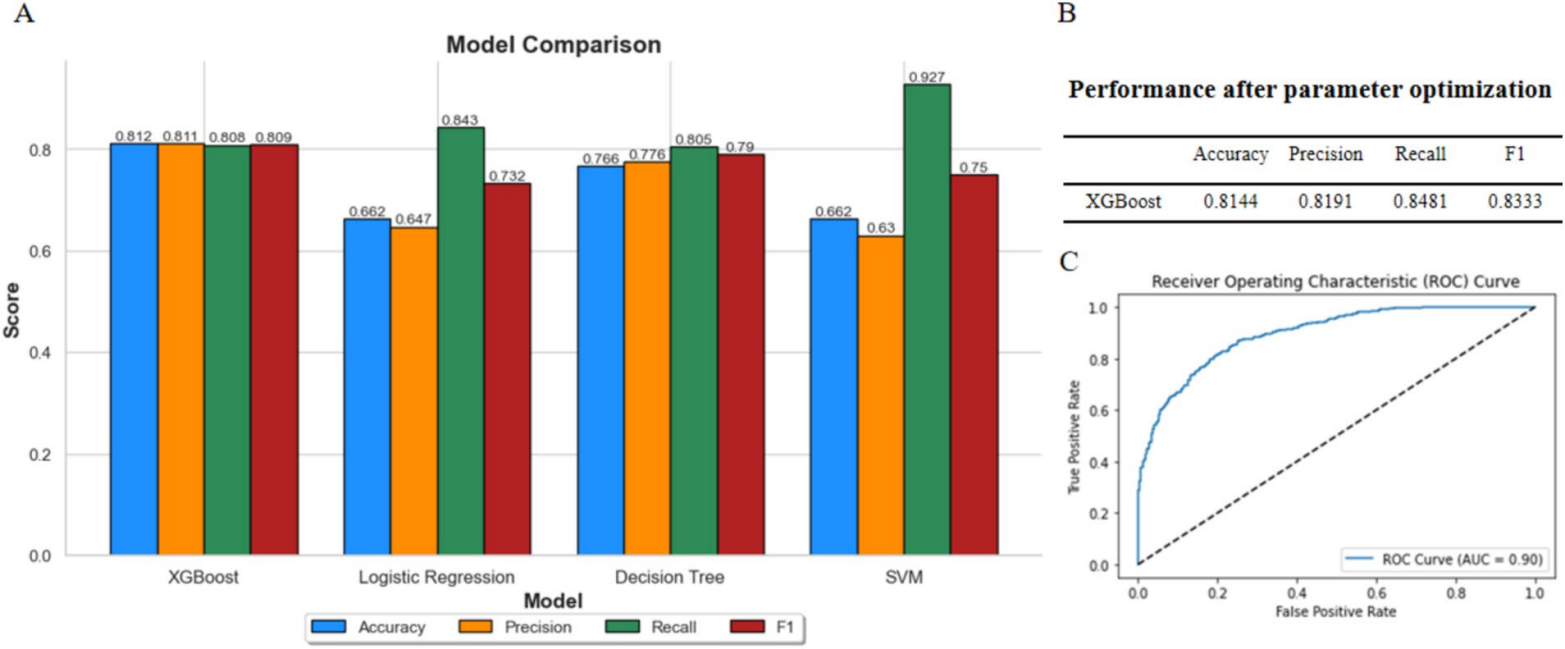
Comparison of model performances. **(A)** Comparison of XGBoost, logistic regression, decision tree, and SVM. **(B)** Performance of XGBoost after parameter optimization. **(C)** ROC curve of XGBoost model.

Using BMI classification as the response variable and incorporating the 40 bacterial species listed in Table 1, along with gender and age as feature variables, we constructed prediction models using XGBoost, logistic regression, decision tree, and support vector machine (SVM). The results represent the average performance metrics (accuracy, precision, recall, *F*_1_-score, and AUC) obtained from 5-fold cross-validation on the validation data. Among these models, the XGBoost model achieved the highest overall performance (Figure 3A), demonstrating its suitability for this task.

Subsequently, we used GridSearchCV to fine-tune the parameters of the XGBoost model. The performance metrics and ROC curve of the optimized model are shown in Figures 3B,C. The fine-tuned model achieved an accuracy of 0.8144, a precision of 0.8191, a recall of 0.8481, and an *F*_1_-score of 0.8333. Additionally, the AUC was 0.90, indicating that the model performs well and is suitable for further research.

### Model global visualization

**Figure 4:**
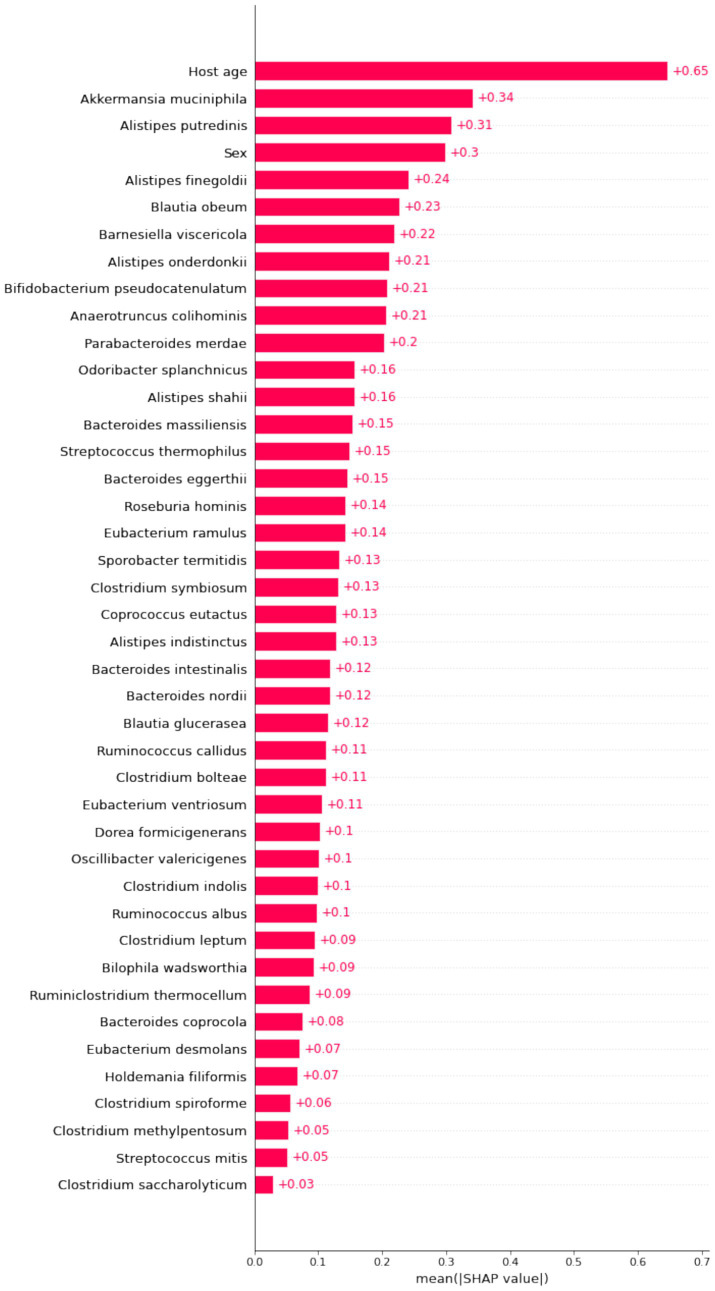
Importance matrix plot of the XGboost model. This importance matrix plot depicts the importance of each covariate in the development of the final predictive model.

The importance matrix of the XGBoost model, shown in Figure 4, highlights the top 10 most significant variables contributing to the model. These variables are host age, *Akkermansia muciniphila*, *Alistipes putredinis*, sex, *Alistipes finegoldii*, *Blautia obeum*, *Barnesiella viscericola*, *Alistipes onderdonkii*, *B. pseudocatenulatum*, and *Anaerotruncus colihominis*.

**Figure 5:**
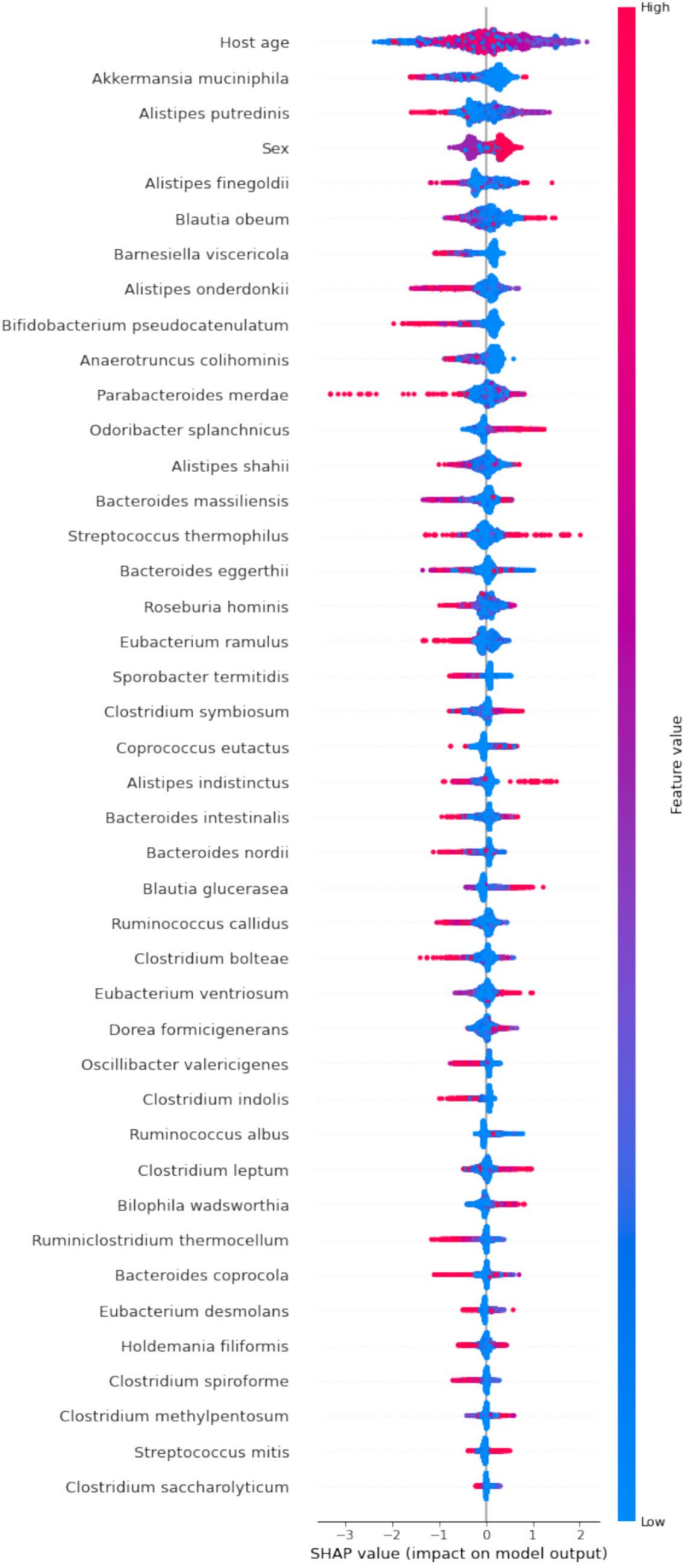
SHAP summary plot of the XGBoost model. Each dot in the plot represents a feature attribution value for a patient, with one dot per feature per patient. Dots are colored based on the feature values for each patient, with red indicating higher feature values and blue indicating lower feature values. The vertical accumulation of dots illustrates the density of feature attributions.

By calculating the Shapley values for each feature, we gain a clear understanding of how each feature influences the model’s predictions. This is crucial for revealing feature importance and the predictive mechanisms of the model (Ning et al., 2022). To identify the features with the most significant impact on the predictive model, we plotted the SHAP summary plot for the XGBoost model (Figure 5). Higher SHAP values for a feature indicate a greater likelihood of being overweight.

Our analysis revealed that features such as *Akkermansia muciniphila*, *Barnesiella viscericola*, *Alistipes onderdonkii*, *B. pseudocatenulatum*, and *Anaerotruncus colihominis* have negative SHAP values, suggesting that their abundance is inversely related to BMI. In contrast, *Odoribacter splanchnicus* exhibits a positive impact on BMI. While features like age and sex significantly contribute to the model’s predictions, their biological implications are less clear in terms of explanatory power.

### Interpretation of personalized predictions

**Figure 6:**
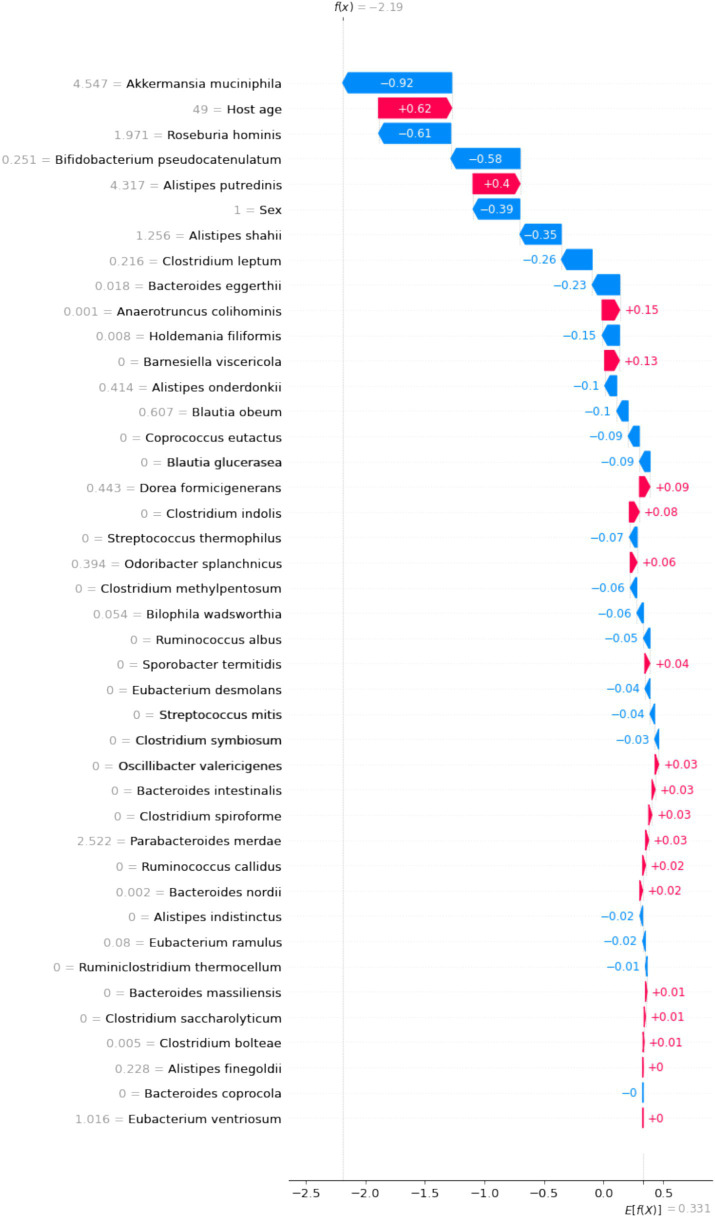
The interpretation of model prediction results with the overweight sample.

SHAP values illustrate the contribution of each feature to the final prediction, providing a clear explanation of the model’s predictions for individual patients. As shown in Figure 6, we selected an overweight sample to demonstrate the model’s interpretability. In this case, age had the highest positive contribution to BMI, with a SHAP value of 0.62. Conversely, *B. pseudocatenulatum*, *Roseburia hominis*, and *Akkermansia muciniphila* had negative contributions to BMI, with SHAP values of −0.58, −6.1, and −0.92, respectively.

### *Bifidobacterium pseudocatenulatum* inhibits fat deposition in *Caenorhabditis elegans*

This study employed machine learning (ML) to predict several bacterial strains associated with BMI. To validate the potential functions of these strains, we examined the effect of *B. pseudocatenulatum* on fat reduction using *Caenorhabditis elegans* as a model, with *Escherichia coli* OP50 as the blank control. Four strains of *B. pseudocatenulatum* (JXL-01, JXL-02, JXL-03, and JXL-05) were selected based on their availability and prior verification of their fat-reducing properties, which were identified through our ML analysis of bacterial strains associated with BMI. Additionally, these strains have been implicated in obesity-related studies (Cano et al., 2013), providing further justification for their selection. These initial findings allowed us to identify the most effective strains for further cell experiments, and lipid accumulation in *C. elegans* was compared after feeding with each of these four *B. pseudocatenulatum* strains.

**Figure 7:**
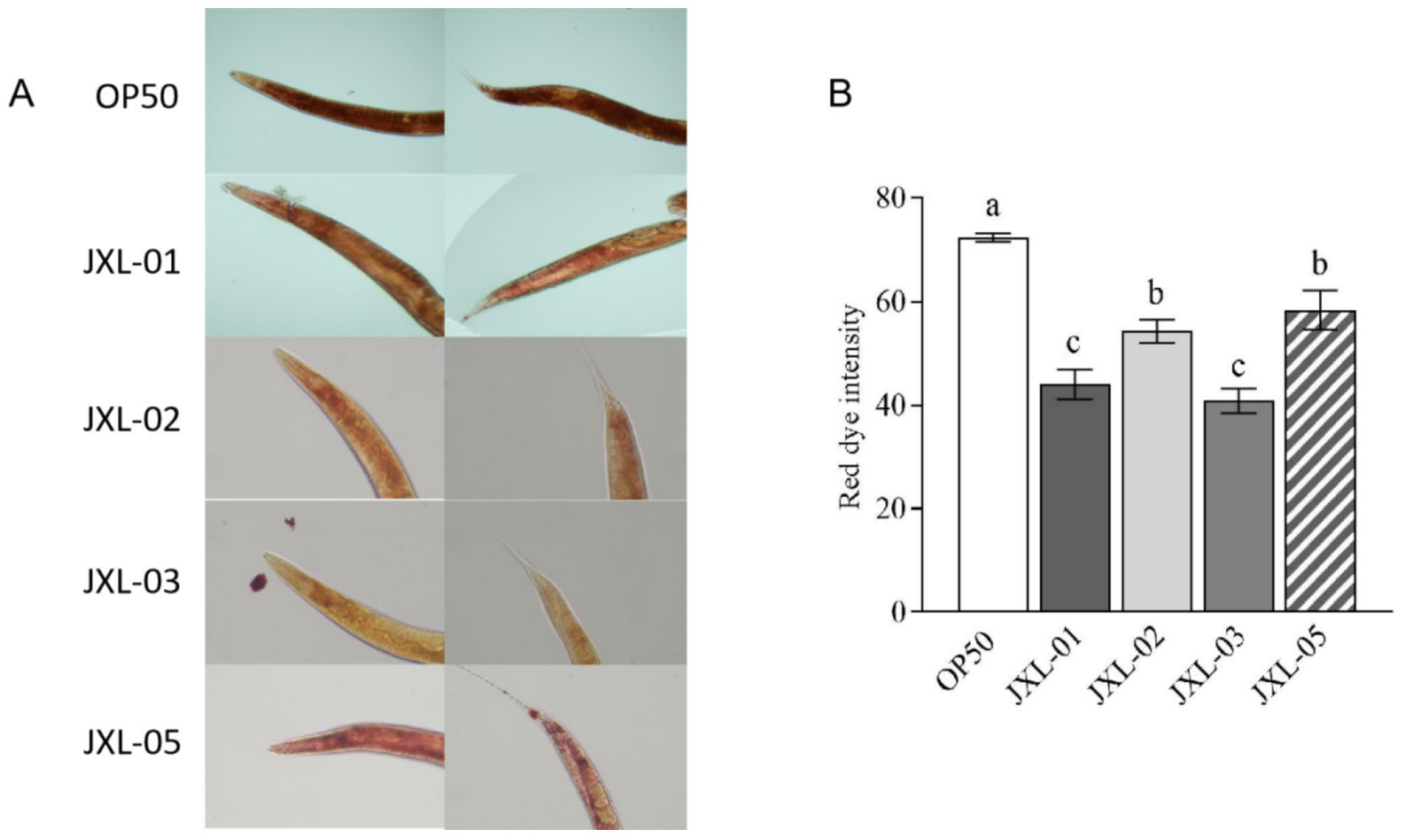
Effect of *B. pseudocatenulatum* on fat metabolism in nematodes. **(A)** Oil Red O staining of Caenorhabditis elegans *C. elegans* to visualize lipid accumulation. **(B)** Quantitative analysis of Oil Red O staining, showing the degree of lipid deposition in the nematodes.

Results showed that, compared to the *E. coli* OP50 group, fat content in nematodes fed with the four strains of *B. pseudocatenulatum* was significantly reduced (Figure 7A). Further analysis of red fluorescence intensity, which indicates fat content, revealed that the strains JXL-01, JXL-02, JXL-03, and JXL-05 resulted in significant reductions in fat fluorescence intensity by 39.25, 24.95, 38.6, and 19.29%, respectively (Figure 7B). These findings suggest that *B. pseudocatenulatum* can significantly reduce fat deposition in nematodes, thereby influencing fat metabolism.

### *Bifidobacterium pseudocatenulatum* inhibits adipogenic differentiation

**Figure 8:**
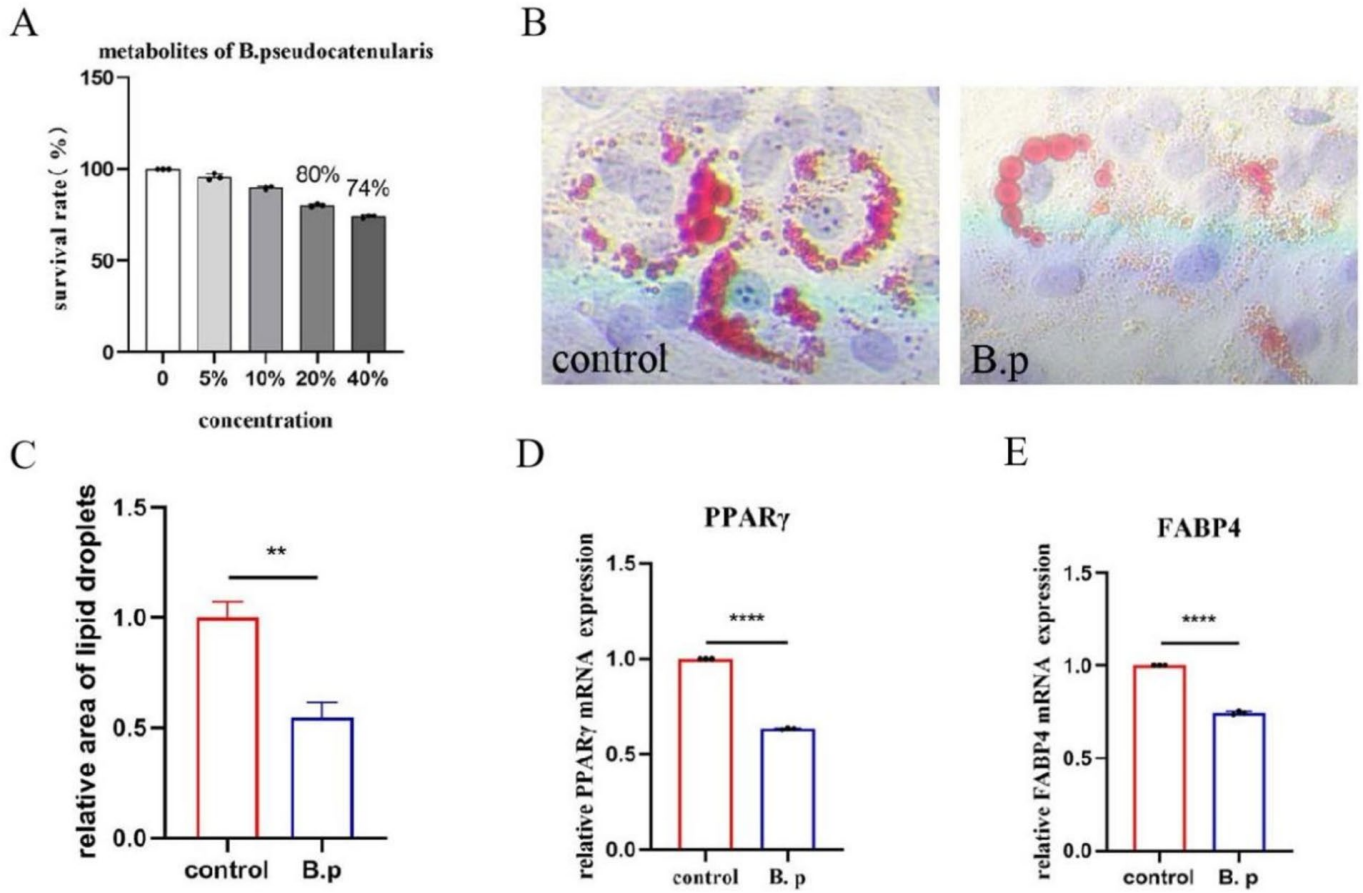
Effect of *B. pseudocatenulatum* on adipogenic differentiation of C3H10 Cells. **(A)** Survival rate of C3H10 cells. **(B)** Oil Red O staining. **(C)** Relative area of lipid droplets. **(D)** Relative PPARγ mRNA expression. **(E)** Relative FABP4 mRNA expression.

Based on the results from the *C. elegans* experiment, we selected the *B. pseudocatenulatum* JXL-01 strain for further investigation into its effect on adipogenic differentiation using mouse mesenchymal stem cells (C3H10 cells). The cells were divided into two groups: a control group and a *B. pseudocatenulatum* intervention group (B.p). To assess the impact of *B. pseudocatenulatum* metabolites on cell viability, a CCK8 assay was first performed. We chose to co-culture C3H10 cells with 10% of the bacterial metabolites (Figure 8A). To evaluate the effects on adipogenic differentiation, we used Oil Red O staining to visualize lipid accumulation. The results showed that lipid droplets were significantly reduced in the B.p group compared to the control group (Figure 8B). Quantitative analysis of the lipid content confirmed the reduction in lipid droplets in the B.p group (Figure 8C). Furthermore, we assessed the expression of key adipogenic transcription factors, including PPARγ and FABP4, which are upregulated during adipocyte differentiation. The mRNA levels of PPARγ and FABP4 were significantly inhibited in the B.p group (Figure 8D–E), suggesting that *B. pseudocatenulatum* can significantly reduce adipogenic differentiation. These findings demonstrate that *B. pseudocatenulatum* JXL-01 significantly influences fat deposition and adipogenic differentiation in C3H10 cells, promoting a reduction in fat accumulation.

## Discussion

Obesity represents a significant public health challenge (Artasensi et al., 2023). The role of the microbiome in health and disease is increasingly recognized, yet the complex and dynamic interactions between host-microbiome and microbe-microbe interactions complicate the identification of beneficial versus harmful bacteria or bacterial combinations. While many studies have reported correlations between the microbiome and health (Van Hul and Cani, 2023), large-scale data studies on obesity-related microbiomes utilizing ML are lacking.

In this study, we predicted the distribution of gut microbiota between overweight (BMI ≥25) and normal weight (BMI <25) groups, employing four ML algorithms for classification. Among these, the XGBoost model demonstrated superior performance, achieving an AUC of 0.90. Furthermore, through the use of SHAP values and SHAP plots, we illustrated that ML methods can elucidate the key features of the gut microbiota associated with overweight populations. This approach facilitated the visual interpretation of feature importance, offering a clear understanding of the critical features identified by the XGBoost model.

However, it is important to note a limitation in our study design. Individuals with obesity (BMI ≥30) were included in the overweight group, which was defined as BMI ≥25. This broad definition may mask specific microbiota differences between overweight and obese individuals, potentially limiting the granularity of our findings. Future research should consider analyzing subgroups within the BMI ≥25 category to better delineate microbiota changes associated with varying degrees of overweight and obesity.

Overall, this study makes several important contributions. We employed XGBoost to develop a ML classification model for identifying overweight individuals and confirmed the effectiveness and reliability of XGBoost in this context. Notably, our analysis revealed that the relative abundances of *A. muciniphila*, *B. viscericola*, *A. onderdonkii*, *B. pseudocatenulatum*, and *A. colihominis* are negatively correlated with increased BMI.

Among these gut bacteria, a deficiency or reduced abundance of *A. muciniphila* has been associated with various diseases, including obesity, diabetes, hepatic steatosis, and inflammation, suggesting its potential as a probiotic for improving fat accumulation and treating obesity (Cani et al., 2022). *B. viscericola* has been linked to improved outcomes in obesity and hepatic steatosis in animal studies, with increased abundance of *Barnesiella* species showing benefits (Rodriguez et al., 2020). Alistipes is a relatively new genus, and human studies have found that *A. onderdonkii* is associated with weight loss following fecal microbiota transplantation and is negatively correlated with waist circumference (Zhang et al., 2022; Qin et al., 2021). Although research on *B. pseudocatenulatum* is limited, available studies suggest that supplementation with this bacterium can improve obesity-related bone metabolism disorders and regulate inflammation associated with obesity (Fernandez-Murga et al., 2020; Sanchis-Chorda et al., 2019; Moya-Perez et al., 2015). *A. colihominis*, a bacterium capable of producing short-chain fatty acids, may play a role in improving glucose tolerance (Yao et al., 2020). These findings underscore the potential of specific gut bacteria in managing and understanding obesity, highlighting the value of microbiome research and ML models in identifying key bacterial species linked to health outcomes.

However, it is important to note that training performance metrics, such as accuracy and precision, do not fully reflect a model’s performance in real-world applications. These metrics may be overly optimistic, particularly for high-capacity models like XGBoost, which can fit well to training data but may not generalize effectively to unseen data. To obtain a more reliable evaluation of our model’s performance, we relied on cross-validation results, which provide a more robust assessment of the model’s generalization ability.

Based on the findings mentioned, we selected *B. pseudocatenulatum* for further validation of its fat-reducing effects in nematodes and cell models. Our research results indicate that all four strains of *B. pseudocatenulatum* improved lipid deposition in *Caenorhabditis elegans*. Additionally, our studies revealed that the metabolites of *B. pseudocatenulatum* can inhibit the adipogenic differentiation of mesenchymal stem cells. This research not only represents a significant advancement in the application of ML but also provides novel insights into the development of probiotics like *B. pseudocatenulatum* for obesity management.

The gut microbiota is believed to play a significant role in the pathophysiology of obesity and is considered a potential therapeutic target. Bacterial metabolites, including short-chain fatty acids (SCFAs), bile acids, and trimethylamine N-oxide (TMAO), interact with specific receptors such as peroxisome proliferator-activated receptors *α* (PPARα) and γ (PPARγ), aryl hydrocarbon receptor (AhR), and G-protein-coupled receptors (GPR41, GPR43, GPR119, TGR5), thereby influencing host metabolism (de Vos et al., 2022; Bai et al., 2020). This underscores the need for further research to develop probiotic products and to better understand the mechanisms of microbe-host interactions.

## Conclusion

The gut microbiota is closely associated with overweight and obesity. By employing ML methods, we developed a classification prediction model to identify and screen gut microbiota related to overweight. The integration of ML and SHAP (SHapley Additive exPlanations) offers clear explanations for individualized risk predictions, allowing researchers to intuitively understand the impact of key features in the model. Our analysis identified several key bacteria negatively correlated with obesity, including *Alistipes onderdonkii*, *Bacteroides massiliensis*, *Haemophilus parainfluenzae*, and *B. pseudocatenulatum*.

Further *in vivo* and *in vitro* experiments have demonstrated that *B. pseudocatenulatum* may influence fat deposition in nematodes and adipocyte differentiation. These findings suggest that it could be a promising intervention for weight loss. Overall, our study represents a significant and necessary advancement toward utilizing gut microbiota modulation or specific active compounds for weight reduction.

## Data Availability

The original contributions presented in the study are included in the article/Supplementary material, further inquiries can be directed to the corresponding authors.

## Footnotes

^1^ https://gmrepo.humangut.info
